# Genetic diversity of major histocompatibility complex class I genes in *Zootoca vivipara*

**DOI:** 10.1042/BSR20193809

**Published:** 2020-04-24

**Authors:** Wanli Liu, Yufen Liu, Peng Liu, Wenge Zhao

**Affiliations:** College of Life Science and Technology, Harbin Normal University, Harbin 150025, P.R. China

**Keywords:** genetic diversity, major histocompatibility complex, Zootoca vivipara

## Abstract

The Major Histocompatibility Complex (*MHC*), as a family of highly polymorphic genes associated with immunity in the genome of the vertebrate, has become an important indicator for assessing the evolutionary potential of wildlife. In order to better protect *Zootoca vivipara* in the Greater Khingan Range and Lesser Khingan Range, to understand the genetic structure of *Z. vivipara*, and to explore the mechanism and phylogenetic relationship of the gene polymorphisms, the *MHC* molecular marker method was used to analyze *Z. vivipara* population. Forty-seven alleles were obtained from four populations. The four populations were highly polymorphic, rich in genetic information, and had significant genetic diversity. There were certain inbreeding phenomena. There was a high degree of genetic differentiation among populations, which was caused by genetic drift and natural selection. The sequence undergoes genetic duplication and recombination. The existence of trans-species polymorphism was found in the constructed phylogenetic tree. The present study provides a theoretical basis for species protection of *Z. vivipara*.

The Major Histocompatibility Complex (*MHC*) is a highly polymorphic receptor multigene family in the genome of the vertebrate, which is closely related to the immune system. Due to the need to identify multiple pathogens and adapt to the environment, *MHC* genes are polygenic and polymorphic, and are related to the survival and reproduction of the population, as well as immunity against disease. This is the unique advantage of *MHC* in population genetics and conservation genetics. Therefore, *MHC* has been considered to protect the latest genetic systems in genetics research. *MHC* molecular markers can overcome such limitations as maternal inheritance of mitochondrial genes and time consumption of microsatellites, so that *MHC* genes have become a hot genetic marker in population genetic structure and gene variation analysis, and have a broad application prospect in species conservation genetics [1,2]. Since the characteristics of *MHC* class I genes of *Nerodia sipedon* and *Ameiva ameiva* were first reported in 1992, research on reptile *MHC* genes has been increasing year by year [3]. Researchers often use *MHC* to study endangered animals and new species. For example, in the last decade, there have been many studies on *Sphenodon punctatus*. The researchers successfully obtained sequence information and analyzed their genetic structure and population dynamics [4–7]. Later, when analyzing the *MHC* of some endangered or newly discovered species [8,9], people further studied from the aspects of species relationship, survival and reproduction, and disease resistance [10–17].

*Zootoca vivipara* is a typical representative of the viviparous animals of the lizard family [18]. It is also a representative of the Palaearctic realm lizards [19]. *Z. vivipara* originated in the Mediterranean region. After the Glacial period, the natural selection made life more suitable in high latitudes, cold areas. *Z. vivipara* in China are all oviparous reproductive mode, distributed in Heilongjiang, Inner Mongolia and Xinjiang [20]. Its distribution range is relatively narrow, the population is small, and the distribution area is special. It is the southern limit of the distribution of the species, and the geographical interval of the distribution is large, which has certain research and protection significance. In recent years, with the development of molecular biology, the research on *Z. vivipara* has been deepened. In the origin and evolution of species, the discussion of the double-breeding model of oviparous-viviparous is a hot topic. Different genetic analysis has caused several differences [21–26]. At the same time, the use of molecular marker technology to explore sequence genetic polymorphism is quite concerned by researchers [27–33]. However, *MHC* molecular studies of *Z. vivipara* have not been reported.

Due to the destruction of the ecological environment caused by human disturbance, the habitat of the global *Z. vivipara* has been shrinking, and the number of *Z. vivipara* has gradually decreased [34]. At present, several populations in China are already in the Near Threatened status [35]. Therefore, using *MHC* genes to study the four geographic populations of *Z. vivipara* in China can better describe the *Z. vivipara* gene polymorphisms and population genetic dynamics, and further explain the effects of natural environment and human factors on phylogenetic relationships and existing distribution characteristics. By exploring its genetic polymorphism formation mechanism, the survival status and fitness of the *Z. vivipara* population can also be speculated immunologically. Furthermore, it reveals the evolutionary history of *Z. vivipara* population and the formation mechanism of systematic geographical pattern in the study area. This also provides theoretical support for the scientific protection of *Z. vivipara* species in the next step.

## Materials and methods

### Sample collection and molecular techniques

Thirty-four adult *Z. vivipara* males and females were randomly captured by hand-hunting method from four locations of the east and west of the Greater Khingan Range and Lesser Khingan Range: Ya Keshi county, Inner Mongolia autonomous region; Hu Ma county, Nen Jiang county and Sun Wu county, Heilongjiang province. The lizard’s tail tissue was collected and stored in liquid nitrogen, then the lizard was released back to its original site. Genomic DNA was extracted using the SanPrEP column animal genomic DNA extraction kit of Sangon Biotech (Shanghai) Co., Ltd., and total RNA was extracted by Takara’s TRIzol reagent. Three pairs of primers IMHCF/IMHCR, 2MHCF/2MHCR, and MHCIF/MHCIR were utilized to clone the mRNA fragment of *MHC I*, the DNA fragment of *MHC I* α2, and the DNA fragment of *MHC I* α3 [36]. SSCP analysis and transformation and cloning sequencing were performed separately.

### Allele determination and sequence alignment

Sequencing successful sequence peak maps were read with Chromas 2.6.5 software, and Blast homologous alignments (https://blast.ncbi.nlm.nih.gov/Blast.cgi) were performed on the NCBI webpage to determine positive clones, whether the sequence is a gene. The sequence alignment used Clustalx 2.1 with default parameters. The sequence alignment map was run with DNAMAN 9.0 and the predicted amino acid sequence was translated using MEGA 7.0 [37] and placed in the Expasy reading frame (http://web.expasy.org/translate/). The information of the alleles was acquired by sequence alignment and calculation of the polyacrylamide gel image, SSCP method. The two methods mutually verify that the sequence occurring simultaneously in two or more individuals is judged as an allele [38].

### Calculation of genetic polymorphism

Nucleotide polymorphism (π) and variable sites (S) were calculated by using Dnasp [39]. Popgene 1.32 software was used to calculate the number of alleles (Na), the number of effective alleles (Ne), the expected heterozygosity (He), and the observed heterozygosity (Ho) and the Shannon information index (I). Polymorphic information content (PIC) [40], gene flow (Nm), Inbreeding coefficient (Fis), and population fixation index (Fst) were calculated by using Cervus 3.0 software. Then MEGA was used for Tajima’s D neutrality test. Amino acid sequence alignments were performed by using online COBALT (www.ncbi.nlm.nih.gov/tools/cobalt/). According to the recent *MHC* I gene research, the gene sequence of *Z. vivipara* relatives was downloaded from GenBank and rooted in human *HLA-A* and *HLA-G*. *MHC* gene homology relationship of four populations of *Z. vivipara* was detected by MEGA software. Then, based on the Poisson-based model (Neighbor-joining, NJ), the phylogenetic tree and branches are constructed for its nucleotide and amino acid sequences. The node confidence (BP) was acquired by 1000 repeated sampling using Bootstrap. In addition, the amino acid sequence of *Z. vivipara* relatives was downloaded from GenBank as an outer group, with human *HLA-A, HLA-B, HLA-C, HLA-G*, and *HLA-I*.

### Selection and recombination detection

MEGA software calculated the nucleotide distance (Kimura 2-parameter model, K2P), amino acid distance (Poisson-corrected model), the ratio of non-synonymous mutation rate to synonymous mutation rate (dN/dS), self-expanding repetition value 1000 [41]. The selection site was tested using the Z-test of MEGA to calculate the sequence selection site and was detected using the FEL, MEME, FUBAR model (http://www.datamonkey.org/) [42]. Restructuring tests were performed using the GRAD model on the Datamonkey website (http://www.datamonkey.org/).

## Results and discussion

### Genetic diversity

**Figure 1:**
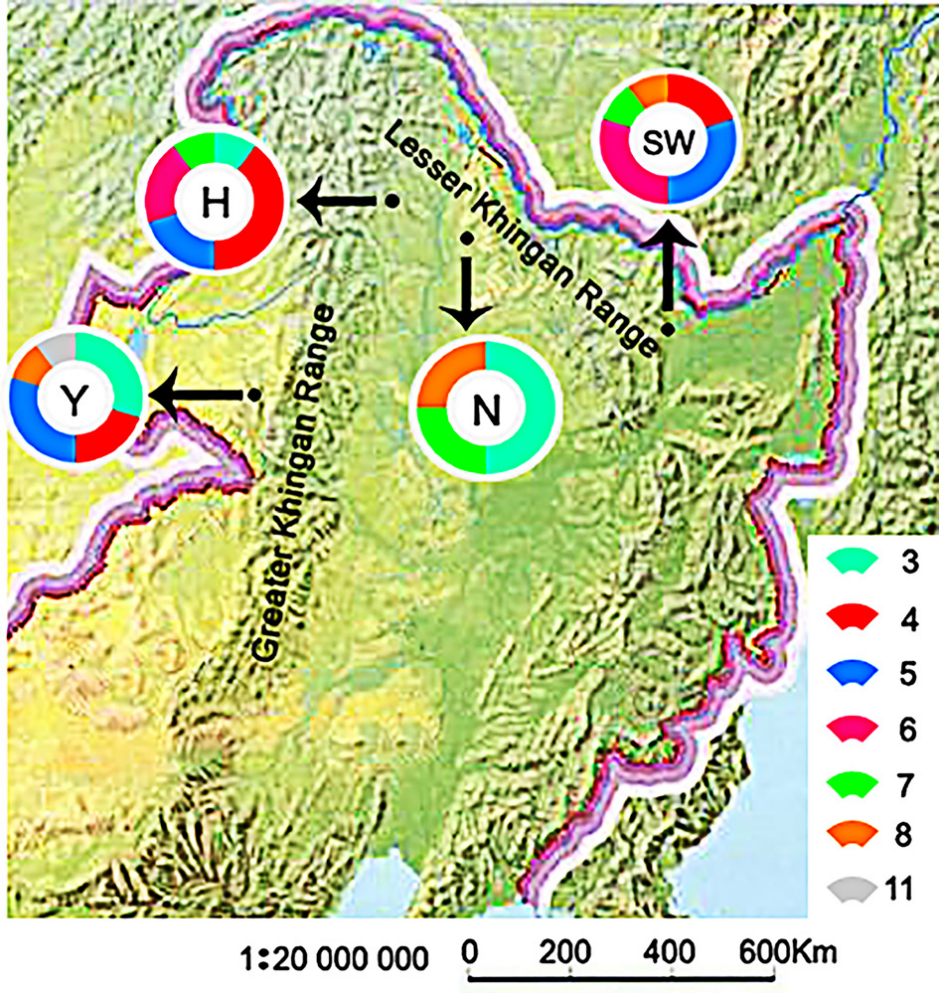
Sample map of *Zootoca vivipara* Pie charts represent the proportion of individuals with different allele numbers in various groups. The population codes are as follows: Y indicates Ya Keshi population; H indicates Hu Ma population; N indicates Nen Jiang population; SW indicates Sun Wu population.

The present study amplified *MHC I* genes of four populations of *Z. vivipara* in China for the first time, and cloned the complete exons 3 and 4 regions from the DNA and RNA molecule level for subsequent analysis. A total of 570 sequences were successfully cloned from 34 individuals of four populations of *Z. vivipara*. After comparison with Blast sequences, they were confirmed to be classical *MHC* class I functional genes [38]. The presence of non-canonical *MHC* class I genes was not found in this study, probably due to the small sample size of four populations and the small number of ten positive clones selected per individual, resulting in the failure to acquire all of the alleles. Among them, the acquired *MHC I* α2 has a nucleotide length of 201–223 bp, α3 has a nucleotide length of 181–184 bp, and α2 and α3 have an amino acid length of 111–178 aa. The number of alleles of four populations of *Z. vivipara* studied in this experiment differed, including 3–11 alleles. So, it was found that the same allele appeared differently in different individuals of different populations (Figure 1), and this difference also reflected the polymorphism of *MHC* class I genes [1,2]. This study used the number and calculation of alleles to speculate on the number of loci per *Z. vivipara* population. The results showed that there were at least three *MHC* I loci in Nen Jiang population (N). There were at least four *MHC* I loci in Sun Wu population (SW). There were at least six *MHC* I loci in Hu Ma population (H) and there were also at least six *MHC I* loci in Ya Keshi population (Y). Among them, the highest π of Y was 0.659, N had a lower π of 0.305, N had the most S in *MHC* class I genes (580), and H had the least S (306) (Table 1). It can be found that the nucleotide polymorphisms of *MHC* class I alleles of four populations of *Z. vivipara* were higher. Due to the current strict computational model, some loci may be ignored. However, the results of the analysis still show that there was a high degree of polymorphism at the locus level of four populations of *Z. vivipara*. It was speculated that *MHC* class I gene in *Z. vivipara* had the phenomenon of gene duplication and gene deletion in the evolution process, which is consistent with the ‘Birth-and-death model of evolution’ [43].

**Table 1:** Nucleotide polymorphism analysis of *MHC* class I alleles of four populations of *Z. vivipara*

Populations	Number of loci	Nucleotide polymorphism, π	Number of variant sites, S
Nen Jiang (N)	3	0.305	580
Sun Wu (SW)	4	0.478	372
Hu Ma (H)	6	0.385	306
Ya Keshi (Y)	6	0.659	529

**Figure 2:**
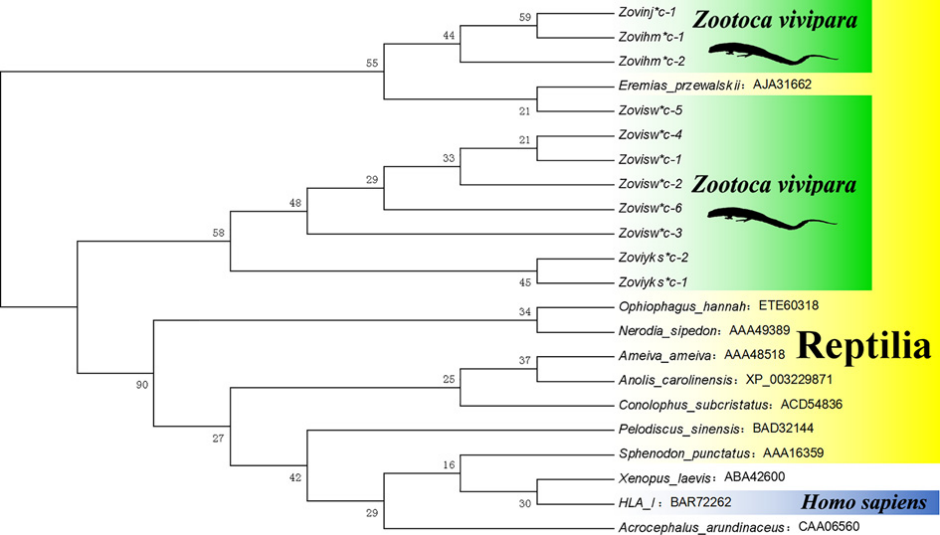
Amino acid phylogenetic tree of *MHC* class I gene of *Z. vivipara* based on NJ method Bootstrap values from 1000 iterations were indicated above the branches.

Alleles were named as *Zovinj, Zovisw, Zovihm*, and *Zoviyks*. The 11 mRNA sequences acquired were translated into amino acid sequences by MEGA software. *MHC* I sequences of 11 other animals were used as the outer group for comparison. When amino acid sequence alignments were performed, it was found that the putative antigen binding sites were all on the α2 domain and the β-2 microglobulin binding site was on the α3 domain. It can be observed in the difference in sequence that the polymorphism of the α2 domain was higher than that of the α3 domain, which may be related to the recognition of multiple antigens and the maintenance of protein structure. Using DANMAN to describe the homology relationship of sequences, it was found that the homology of different alleles in various groups was high. The homology of populations divided four populations into two categories: N and H had high homology, first clustered into one, and then clustered with SW. Finally, they were clustered together with Y, and the geographical distance from the populations were inconsistent. From the phylogenetic relationship, the four populations of *Z. vivipara* were mainly clustered in the NJ tree, and the self-supporting rate was higher, there was no significant difference. In the NJ tree at the DNA level, H was independent of one branch in the α2 domain, and SW was independent of one branch in the α3 domain. Respectively in H and SW appeared to branch, which may be due to the presence of positive and negative sequencing during cloning sequencing. At the same time, these sequences were under the same root, the difference was not significant. Then the phylogenetic analysis of the full amino acid sequence of α2 and α3 domains. It was found that four populations of *Z. vivipara* were clustered into two (Figure 2). This difference may be caused by the different evolutionary relationships between different domains caused by gene recombination [14]. It also indicated that *MHC* class I gene of *Z. vivipara* had high polymorphism as a whole. It is consistent with the previous analysis of the genetic diversity of *Z. vivipara* population in China [31,32]. In the above analysis of various levels of polymorphism, it was found that all four populations were highly polymorphic, which may be related to the ability to resist disease.

### Genetic structure

In all populations, Na was generally greater than Ne. This indicated that alleles were unevenly distributed among the populations. The Ho of populations were generally less than He. This showed that there was a certain genetic variation [44]. PIC was generally greater than 0.5, belonging to the high polymorphic locus [40]. Using the Grant et al. method to estimate the demographic, it was found that the four populations of *Z. vivipara* were the result of long-term differentiation [45]. Compared with other reptilian animals, the polymorphism coefficient was found to be lower than that of the widely distributed species, but higher than several endangered species. It indicated that four populations of *Z. vivipara* may be more interfered, which is consistent with the near threatened status. The gene richness is directly proportional to I. Since *Z. vivipara* has I between 0.5 and 1, the gene was abundant [46]. Most Ho was less than He and Fis was greater than 0. Inbreeding was common within the population, and distant breeding occurred occasionally. This result was consistent with the mating method of *Z. vivipara* and the reduction in the number of individuals in the population. Tajima’s Test of Neutrality was greater than 0 indicating that the population was affected by balanced selection, and population contraction may have occurred, which was consistent with the increased survival risk of *Z. vivipara* populations (Table 2). Fst was generally was greater than 0.25, and there was a high degree of genetic differentiation between the populations [47]. Gene flow is an important factor affecting the genetic structure of the population. Meanwhile, the Nm between *Z. vivipara* populations was less than 1, genetic drift became the dominant factor in the differentiation of the population genetic structure [48], indicating less genetic exchange between the populations (Table 3). It was speculated that the effect of genetic drift causes the four populations of *Z. vivipara* to differentiate, and there may be some decline between the populations [49]. This may be related to habitat destruction and reduction.

**Table 2:** The genetic diversity index of four populations of *Z. vivipara* in *MHC* class I

Populations	Sites	Number of alleles, Na	Number of effective alleles, Ne	Observed heterozygosity, Ho	Expected heterozygosity, He	Polymorphic information content, PIC	Shannon information index, I	Inbreeding coefficient, Fis	Tajima’s D
Nen Jiang (N)	Full-length	9.0000	7.1053	1.0000	0.8947	0.5550	0.7500	−0.1429	1.722
	(α2) Exon3	3.0000	2.4000	0.0000	0.6000	0.4070	0.7000	0.2174	2.650
	(α3) Exon4	3.0000	2.4000	0.0000	0.6000	0.5660	0.6250	0.1128	3.759
Sun Wu (SW)	Full-length	2.0000	1.8000	0.0000	0.3333	0.6670	0.6389	0.3333	4.295
	(α2) Exon3	3.0000	2.5714	1.0000	0.4286	0.4287	0.7000	−0.2903	4.269
	(α3) Exon4	2.0000	1.6667	0.0000	0.3333	0.3333	0.7130	0.3378	3.530
Hu Ma (H)	Full-length	2.0000	1.6667	0.0000	0.6000	0.8330	0.7045	0.2800	0.902
	(α2) Exon3	6.0000	4.8462	1.0000	0.1538	0.7500	0.6974	−0.3097	3.383
	(α3) Exon4	9.0000	7.1053	1.0000	0.8947	0.5858	0.7115	−0.0800	2.360
Ya Keshi (Y)	Full-length	2.0000	1.6667	0.0000	0.6000	0.3860	0.7045	0.0150	2.323
	(α2) Exon3	6.0000	4.6364	0.0000	0.3636	0.6050	0.7130	0.0368	2.922
	(α3) Exon4	3.0000	2.4000	0.0000	0.6000	0.7440	0.7000	0.0474	3.938

**Table 3:** Genetic divergence (below diagonal) and gene flow (above diagonal) among four populations of *Z. vivipara* in the *MHC* class I

Populations	Nen Jiang (N)	Sun Wu (SW)	Hu Ma (H)	Ya Keshi (Y)
Nen Jiang (N)	—	0.8202	0.9912	0.4119
Sun Wu (SW)	0.2835	—	0.7331	0.3807
Hu Ma (H)	0.2302	0.2575	—	0.5878
Ya Keshi (Y)	0.3981	0.4017	0.3202	—

The genetic divergence (below diagonal) and gene flow (above diagonal).

### Positive selection and recombination detection

In the present study, *MHC* class I gene was highly polymorphic, and its causes were analyzed and its maintenance mechanism was speculated. First, the genetic distance analysis showed that the ratio of the average nucleotide distance to the average amino acid distance of the four populations was generally less than 1, and the difference between each gene region was extremely significant (Table 4). Second, the ratio of the non-synonymous mutation rate to the synonymous mutation rate in different gene domains of different populations ω (dN/dS) was generally greater than 1, suggesting that *MHC* I genes of the four populations may be selected (Table 5). Then four methods were used to perform positive selection calculations on four different gene domains. Some amino acid sites that were positively selected existed near the putative antigen binding site [45]. Finally, these analyses indicate that *Z. vivipara*’s *MHC* class I genes were likely to have undergone positive selection. The pathogen-mediated selection of pathogens and parasites is one of the main reasons for maintaining high polymorphism, indicating that the positive selection was highly polymorphic with *MHC* gene of *Z. vivipara* and the regulation of pathogens by *Z. vivipara* (Table 6). Since the degree of variation of the antigen binding site is positively related to the type of antigen that can be recognized, which plays an important role in antigen recognition and presentation in the body’s immune response. This is similar to other vertebrate immune mechanisms [6,41,50]. *Z. vivipara* likes to live in a hidden environment, where it is mostly dark and humid, breeding many parasites and pathogens. This situation may be the reason why *Z. vivipara* was receiving positive selection. However, some positive selection mutation sites are not on the putative antigen-binding region, which may also be related to some important sites different from humans. The habitat of *Z. vivipara* is subject to certain human disturbances, and this effect may also alter the outcome of parasite-mediated selection [17].

**Table 4:** *MHC* I genetic divergence of nucleotide and amino-acid within four populations of *Z. vivipara*

Populations	Ave. nucleotide divergence/Ave. amino-acid divergence		
	Full-length	(α2) Exon3	(α3) Exon4
Nen Jiang (N)	0.314	0.606	0.470
Sun Wu (SW)	0.210	0.417	0.679
Hu Ma (H)	0.350	0.873	0.344
Ya Keshi (Y)	0.406	0.458	0.380

**Table 5:** Ratio of non-synonymous mutation rate to synonymous mutation rate

Populations	Sites	Non-synonymous, dN	Synonymous, dS	(ω) dN/dS	*P*-value
Nen Jiang (N)	Full-length	0.291	0.303	0.960	0.020
	(α2) Exon3	0.661	0.339	1.949	0.016
	(α3) Exon4	0.667	0.333	2.000	0.060
Sun Wu (SW)	Full-length	0.285	0.283	1.007	0.050
	(α2) Exon3	0.726	0.274	2.650	0.019
	(α3) Exon4	0.769	0.231	3.333	0.048
Hu Ma (H)	Full-length	0.152	0.149	1.020	0.069
	(α2) Exon3	0.706	0.294	2.406	0.011
	(α3) Exon4	0.707	0.293	2.412	0.021
Ya Keshi (Y)	Full-length	0.349	0.259	1.347	0.028
	(α2) Exon3	0.887	0.113	7.875	0.013
	(α3) Exon4	0.686	0.314	2.182	0.020

The difference is significant when *P*<0.05.

**Table 6:** Positive selection site analysis of *Z. vivipara*

Gene sites	Method	Sites predicted to be under positive selection																																		
Full-length		2	4	5	6	7	9	10	11	14	15	17	23	24	28	29	30	34	35	40	41	43	44	56	60	64	74	85	104							Sum
	MEGA							+		+		+	+		+			+		+			+	+	+	+										11
	FEL		+											+						+																3
	MEME	+	+	+	+	+	+		+	+	+			+	+	+	+	+	+	+	+	+					+	+	+							21
	FUBAR		+																																	1
(α2) Exon3		1	2	3	4	5	6	7	8	9	11	12	14	16	17	18	20	25	26	33	37	39	40	41	42	43	44	46	49	50	51	54	60	61	63	Sum
	MEGA	+		+	+	+	+	+	+	+	+	+	+	+	+	+	+	+	+	+	+	+	+	+	+	+	+	+	+	+	+					29
	FEL																					+														1
	MEME		+													+																	+	+	+	5
	FUBAR																															+				1
(α3) Exon4		10	15	17	21	22	23	25	27	28	30	32	34	35	39	40	41	44	53	61	72	100														Sum
	MEGA	+	+	+	+	+	+	+		+			+	+	+	+	+	+																		14
	FEL									+									+		+															3
	MEME								+		+	+										+														4
	FUBAR																			+																1

Recombinant analysis of *MHC* I gene sequences of four populations of *Z. vivipara* were performed using online software, and traces of three recombination effects (81, 149, and 161 points) were detected in the *MHC* sequence. And the difference was extremely significant (*P*<0.01). Recombination of *MHC* genes behaves differently in different species of vertebrates. In Osteichthyes and Amphibians, exon shulffing is caused by different evolutionary relationships of different domains [51]. In the study of the birds *MHC* gene, it is found that gene recombination and transformation occurred intralocus [52,53]. While in mammals only mini-casettes are recombined [54,55]. This difference is due to the different stages of the evolution of *MHC I* gene. Intralocus recombination, especially allelic recombination, is often found in reptiles *MHC*, which can produce high polymorphism of *MHC* and maintain it. According to the reported sequence characteristics of *MHC* gene in geckos, it is found that the polymorphism of *MHC* gene mainly comes from gene recombination [56]. There was also a gene recombination event in the high polymorphism *MHC* sequence of *Ctenophorus decresii* [14]. In this study, three genetic recombinations were found in the *MHC* sequence of *Z. vivipara* using the GRAD model, which was located between exon 3 and exons 3 and 4. It indicates that the sequence had been broken and the gene exchange during the evolution process. Therefore, genetic recombination also plays a role in maintaining the polymorphism of the *MHC* class I gene in *Z. vivipara*. However, due to the current lack of certain rules for the genetic recombination of non-mammalian *MHC* class I genes, different environmental factors and gene structures of different species may lead to different genetic recombination results. Moreover, no recombination phenomenon was found in the analysis of three mitochondrial gene regions and three nuclear gene loci in the Eurasian region [33], and it is not possible to determine whether this recombination event was prevalent in all populations of *Z. vivipara*. Finally, the small population size, number of individual samples, and number of alleles in the present study may also limit the discovery of recombination phenomena.

### Cross-species polymorphism analysis

The phylogenetic NJ tree of *MHC I* α2 and α3 full amino acid sequences was constructed by using the amino acid sequence used in the sequence alignment as exogroups (Figure 2). The phylogenetic NJ tree of *MHC* I α2 and α3 was constructed by using the 22 related sequences downloaded from NCBI acquired as an outer group. The study found that the four populations of *Z. vivipara* were clustered from the phylogenetic tree of *MHC I* mRNA. So, there was no obvious differentiation of four populations of *Z. vivipara*, indicating that there were shared alleles between them. This is consistent with the results of previous genetic polymorphism. In addition, in the three phylogenetic trees, *MHC I* gene sequence of the *Eremias przewalskii* always clustered with *Z. vivipara MHC I* gene sequence. The examination of the whole experiment and analysis process was confirmed to be correct, indicating that there was a cross-species polymorphism in *Z. vivipara MHC I* gene. The species belonged to *Zootoca spp*. and *Eremias spp*. The phylogenetic relationship in the phylogenetic tree was greater than that of *MHC I* gene system, suggesting that *MHC I* gene already existed at least before the differentiation of *Z. vivipara* and *E. przewalskii.* The species differentiation of *Z. vivipara* has been identified as 4.5 million years ago (95% confidence interval: 2.6–6.1 million years ago) [22], and the species differentiation of *Eremias spp*. has been identified as 6.3 million years ago (95% confidence interval: 5.3–8.5 million years ago) [57]. It was speculated that *Z. vivipara MHC I* allele lineage was formed at least 6 million years ago. Therefore, it may also be related to the adaptation of the lizards to the environment of high latitude and high cold regions in the evolution process [58]. This situation is consistent with the theory that *MHC* is the original gene that appeared before the formation of existing species [59].

In the present study, experiments were performed on *MHC* genes of 34 *Z. vivipara* individuals in four populations. The results showed that four populations of *Z. vivipara* in China had high genetic polymorphisms. The gene replication and gene recombination experienced by *MHC* genes during evolution were maintained by positive selection. At the same time, *Z. vivipara* had inbreeding in the population, and gene exchange and higher degree of genetic differentiation had occurred between the populations by the combination of genetic drift and natural selection. The *MHC* gene of *Z. vivipara* even had trans-species polymorphism. Although the genetic polymorphism of the *Z. vivipara MHC* gene and its formation mechanism had been analyzed in the present study, the immunological mechanism of the *MHC* gene family has not been fully revealed. Since the conservation of *Z. vivipara* is continuing, the molecular immunology of *Z. vivipara* also needs to be further analyzed.

## Abbreviations

MHC: major histocompatibility complex
PIC: polymorphic information content
SSCP: single strand conformation polymorphism
